# Attitudes Toward Vital Signs Assessment as a Predictor of Attitudes Toward Pain Assessment Among Nursing Students With Clinical Practice Experience

**DOI:** 10.1155/prm/7428206

**Published:** 2026-05-27

**Authors:** Burcu Nal, Necibe Dağcan Şahin, Gamze Ünver

**Affiliations:** ^1^ Department of Fundamentals of Nursing, Faculty of Health Sciences, Kütahya Health Sciences University, Kütahya, Türkiye; ^2^ Department of Internal Medicine Nursing, Faculty of Health Sciences, Kütahya Health Sciences University, Kütahya, Türkiye

**Keywords:** attitudes, nursing students, pain assessment, predictors, vital signs assessment

## Abstract

**Purpose:**

This study aimed to examine nursing students’ attitudes toward vital signs assessment and pain assessment by demographic characteristics and to identify the effect of attitudes toward vital signs assessment on attitudes toward pain assessment as well as the predictors of attitudes toward pain assessment.

**Methods:**

A descriptive and analytical cross‐sectional study was conducted between March and June 2025 with 332 second‐, third‐, and fourth‐year nursing students who had clinical practice experience. Data were collected using a Sociodemographic Information Form, the Attitudes Towards Vital Signs Monitoring Scale‐Turkish Version (V‐Scale‐TR), and the Nursing Students’ Attitudes Scale Toward Pain Assessment (NSASPA). Data were analyzed using descriptive statistics, Mann–Whitney U and Kruskal–Wallis tests, Pearson correlation analysis, and multiple linear regression analyses.

**Results:**

A moderate positive correlation was found between nursing students’ attitudes toward vital signs assessment and their attitudes toward pain assessment (*p* < 0.05). Multiple linear regression analysis revealed that demographic variables and attitudes toward vital signs assessment together explained 43.5% of the variance in attitudes toward pain assessment (*R* ^2^ = 0.435, *p* < 0.001).

**Conclusions:**

More positive attitudes toward vital signs assessment were associated with more positive attitudes toward pain assessment. These findings suggest that competence and awareness in vital signs monitoring may enhance nursing students’ sensitivity, empathy, and clinical judgment in pain assessment.

**Implications for an International Audience:**

Integrating evidence‐based education on vital signs assessment and pain assessment into undergraduate nursing curricula may improve nursing students’ critical thinking skills, empathy, and overall quality of patient care across diverse healthcare settings.

## 1. Introduction

Monitoring and reporting of vital signs enables early recognition of deterioration in the clinical picture of patients and timely interventions [1]. Measurement, analysis, regular monitoring, and appropriate reporting of vital signs are among the main responsibilities of nurses [2]. Nurses contribute to the areas of obtaining objective data that will form the basis of nursing care, defining the health problem correctly, and deciding on timely and appropriate approaches to the clinical course of the individual by using the vital signs assessment skills acquired during their basic education [3, 4]. Nursing students have an important potential as future professional practitioners of the nursing profession [5]. Monitoring vital signs as a non‐invasive procedure is one of the nursing practices that nursing students perform independently or require the least supervision during clinical practice [2]. Another factor that affects nursing students’ fulfillment of their roles related to monitoring and recording vital signs is their attitudes toward this issue [2]. The importance of vital signs monitoring as a priority in patient care influences the successful fulfillment of this role [6–8]. Therefore, it is extremely important to identify and understand the factors affecting attitudes toward vital signs. Both vital signs assessment and pain assessment are integral components of fundamental patient evaluation, requiring continuous clinical monitoring and timely decision‐making in order to ensure early intervention and patient safety, particularly in fast‐paced clinical environments [9].

One of the most important parts of the nursing care process is effective pain assessment [10]. Reduction of pain is an important indicator of quality of care, as it contributes to improved quality of life, reduced complications, accelerated recovery, and increased patient satisfaction [11]. For these reasons, the attitude and knowledge of nurses toward effective pain assessment is a very important issue [12]. Nurses, who spend the most time with patients and constantly interact with them, should play an active role in pain assessment [13]. This issue should be addressed, especially during nursing education [14, 15]. The recognition of pain as the “fifth vital sign” further conceptually aligns pain assessment with traditional vital signs, suggesting that both domains may be perceived within a similar framework of essential physiological and clinical indicators in nursing practice [16]. Nursing students encounter numerous patients experiencing pain and provide care to them during their clinical training. The American Pain Society and the Health Care Organization Accreditation Committee recommend that pain should be considered as a “fifth vital sign” along with pulse, respiration, blood pressure, and body temperature [4]. In the literature, no study examining the relationship between nursing students’ attitudes toward vital signs and their attitudes toward pain assessment was found. However, these two domains are both integral to comprehensive patient assessment and require similar cognitive processes such as observation, prioritization, and clinical judgment [17]. Therefore, it is reasonable to assume that attitudes toward vital signs assessment may be associated with attitudes toward pain assessment, as both reflect students’ approach to essential clinical evaluation responsibilities [2, 18]. Based on this gap, this study aimed to examine nursing students’ attitudes toward vital signs assessment and pain according to their demographic characteristics and to identify the effect of attitudes toward vital signs on attitudes toward pain assessment and the predictors of attitudes toward pain. In addition, the findings are expected to guide nurse educators in understanding students’ attitudes in the evaluation of vital signs and pain and to support further research on this topic.

Research questions:

1. What are the attitudes of nursing students toward vital signs?
2. What are the attitudes of nursing students toward pain assessment?
3. What is the relationship between nursing students’ attitudes toward vital signs and pain assessment?
4. What are the predictors of nursing students’ attitudes toward pain assessment, including demographic characteristics and vital signs assessment subdimensions?

## 2. Methods

### 2.1. Type (Design) of Research

This research was conducted as a descriptive and analytical cross‐sectional study between March 2025 and June 2025. This study was reported in accordance with the STROBE (Strengthening the Reporting of Observational Studies in Epidemiology) guidelines [19].

### 2.2. Place and Time of the Research

The study population consisted of 406 nursing students enrolled in the 2nd, 3rd, and 4th years of a state university’s nursing department in Turkey during the spring semester of the 2024–2025 academic year. Students who were 18 years or older, had completed the *Fundamentals of Nursing* course, and voluntarily agreed to participate were included in the study. Students’ clinical practice was conducted at a single teaching and research hospital. This hospital offers a wide range of specialized healthcare services under a standardized organizational structure. Clinical observations were carried out in various departments, primarily internal medicine, surgery, and pediatrics. The same institutional nursing care protocols are applied in all units of the hospital. This provides a consistent and comparable clinical environment for students to observe and evaluate nursing care related to vital sign assessment and pain assessment. Based on a known population sample calculation with a 5% margin of error and a 99% confidence level, the required sample size was determined as 253. However, the study was completed with 332 participants after excluding 52 students who declined participation, 5 who were absent, and 17 with incomplete questionnaire data. In the nursing department where the research was conducted, instruction on vital signs and pain assessment is provided during the second‐year fall semester within the fundamentals of nursing course. Therefore, first‐year students were excluded from the study sample.

### 2.3. Data Collection

The students who volunteered to participate in the study were asked to fill in the “Sociodemographic Form,” “Attitudes Towards Vital Signs Monitoring Scale‐Turkish Version (V‐Scale‐TR),” and “Nursing Students’ Attitudes Scale Toward Pain Assessment (NSASPA)” between March 2025 and June 2025 by self‐report method. Students take approximately 15 min to complete the form. Data were collected through face‐to‐face surveys in a classroom setting. Participants were informed of the study’s purpose and participation requirements, and the data collection process was based on voluntary participation.

*Sociodemographic form:* It consists of 8 questions about the students’ age, gender, grade level, grade point average (GPA), graduation from high school, vital signs, and the source of information about pain.

It consists of 8 questions regarding the students’ age, gender, grade level, GPA, type of high school graduated from, knowledge of vital signs, and sources of information about pain.

#### 2.3.1. V‐Scale‐TR

The V‐Scale was originally developed by Mok et al. [20] to assess nurses’ attitudes toward the monitoring of vital signs for detecting clinical deterioration in patients. The V‐Scale‐TR is a tool that measures nurses’ attitudes toward assessing vital signs such as pulse, blood pressure, oxygen saturation, and respiration. Although pain is considered the “fifth vital sign,” the V‐Scale‐TR does not directly assess pain‐related evaluations. The Turkish adaptation, validity, and reliability study was conducted by Ertuğ (2018) [21]. The scale includes a total of 16 items grouped into five subdimensions: workload (4 items), technology (4 items), communication (2 items), information (3 items), and basic indicators (3 items). It is a five‐point Likert‐type scale, ranging from *strongly disagree* [1] to *strongly agree* [5]. All items, except for items 5, 8, and 9, are negatively worded and thus reverse‐coded. The possible total score ranges from 16 to 80, with higher scores indicating more positive attitudes toward vital sign monitoring, while lower scores reflect more negative attitudes. Cronbach’s alpha reliability coefficient was reported as 0.71 in the original study [20] and 0.76 in the Turkish adaptation [21]. In the present study, the Cronbach’s alpha value for the V‐Scale‐TR was calculated as 0.728. Although the Turkish validity and reliability study of the V‐Scale was conducted among nurses, the scale has also been used in studies involving nursing students [3, 7], and it was applied to a nursing student sample in the present study.

#### 2.3.2. Nursing Students’ Attitudes Scale Toward Pain Assessment

The scale was developed by Bulut et al. (2022). The Scale of Nursing Students’ Attitudes Towards Pain Assessment consists of 15 items. The students indicated their level of agreement with the items according to a five‐point Likert‐type scale as *strongly disagree* [1], *disagree* [2], *undecided* [3], *agree* [4], and *strongly agree* [5]. The scale consists of two main subdimensions: “significance” and “interest.” The “significance” subdimension comprises 12 negatively worded items related to the significance of pain assessment and is reverse‐scored. The “interest” subdimension includes three positively worded items. The total score ranges from 15 to 75, with higher scores indicating more positive attitudes toward pain assessment. Importantly, this scale does not measure the presence or intensity of pain; rather, it evaluates nursing students’ cognitive and affective attitudes toward the process of pain assessment [15]. As the score obtained from the scale increases, the positive attitudes of nursing students toward pain assessment also increase. Bulut et al. (2022) found the Cronbach’s alpha value of the scale to be 0.918 [15]. In this study, Cronbach’s alpha coefficient of the scale was found to be 0.843.

### 2.4. Analysis of Data

The data were analyzed using the IBM SPSS Statistics 25 software package. A total of 17 students with missing data were excluded from the analysis using listwise deletion, and analyses were conducted on complete cases. Descriptive statistics, including frequency, percentage, mean, standard deviation, and median values, were used to summarize the data. The reliability of the scales employed in the study was evaluated by calculating Cronbach’s alpha coefficients. The assumptions of normality were checked using skewness and kurtosis values. Acceptable limits for assessing the normality assumption were set at −1.5 to +1.5 [22]. For variables meeting the normal distribution criteria, parametric tests such as the independent samples *t*‐test and ANOVA were applied, whereas nonparametric tests (e.g., Mann–Whitney U and Kruskal–Wallis tests) were used for variables that did not meet the normality assumption. Following significant results from multiple group comparisons, Bonferroni post hoc tests were conducted to identify pairwise differences between groups. The relationships among independent variables were examined using Pearson correlation analysis. Sociodemographic variables and the subdimensions of the V‐Scale‐TR (workload, technology, communication, knowledge, and key indicators) were included as independent variables, while NSASPA was treated as the dependent variable in the multiple linear regression analysis.

### 2.5. Ethical Aspects of the Study

Institutional permission was obtained from the Nursing Department of the University (decision dated 12.03.2025, No. 177515), and ethical approval was granted by the Non‐Interventional Clinical Research Ethics Committee (decision dated 27.02.2025, No. 2025/03‐02). This study was conducted in accordance with the principles of the Declaration of Helsinki. Participation in the study was entirely voluntary. All data were collected anonymously, and no identifying information was obtained from participants. Participants were informed that they had the right to withdraw from the study at any time without any consequences. In addition, permission to use the *V-Scale-TR* and the *NSASPA* was obtained from the respective authors.

## 3. Results

### 3.1. Sociodemographic Characteristics of Students (*n* = 332)

Among the 332 nursing students, 73.8% were male and 26.2% were female. Regarding class level, 33.1% were second‐year, 38.3% were third‐year, and 28.6% were fourth‐year students. The majority of participants (78.0%) had a GPA between 3.01 and 3.50. Most students graduated from Anatolian High Schools (74.1%), followed by other types of high schools (13.9%), Imam Hatip High Schools (6.3%), technical and health education high schools (4.8%), and general high schools (0.9%).

In terms of previous training, 41.9% of the participants had received education on pain management, while 58.1% had not. Among those who had received such training, 43.9% had only theoretical instruction, whereas 56.1% received both theoretical and practical training. Similarly, 49.7% of students had prior training on the management of vital signs, while 50.3% did not. Of those trained, 15.8% received only theoretical education, and 84.2% received both theoretical and practical training.

### 3.2. Mean Subscale and Total Scores of the V‐Scale‐TR and NSASPA

The mean score of the V‐Scale‐TR among nursing students was 52.96 ± 7.51 (min: 30, max: 74). Regarding its subdimensions, the mean scores were *Workload* 13.36 ± 3.51 (min: 4, max: 20), *Technology* 12.79 ± 3.37 (min: 4, max: 20), *Communication* 7.78 ± 1.85 (min: 2, max: 10), *Knowledge* 10.35 ± 1.76 (min: 6, max: 15), and *Key Indicators* 8.67 ± 1.96 (min: 3, max: 15). The mean score of the NSASPA was 55.98 ± 8.62 (min: 34, max: 71), with subscale mean scores of *Significance* 46.34 ± 7.29 (min: 27, max: 56) and *Interest* 9.63 ± 2.97 (min: 3, max: 15).

### 3.3. Comparison of V‐Scale‐TR and NSASPA Scores by Nursing Students’ Demographic Characteristics

The comparison of the V‐Scale‐TR and its subdimensions and NSASPA scores according to the sociodemographic characteristics of the students is presented in Table 1.

**TABLE 1:** Comparison of V‐Scale‐TR and NSASPA scores according to demographic characteristics.

	**V-Scale-TR**							**NSASPA**
		**Workload**	**Technology**	**Communication**	**Knowledge**	**Key indicators**	**V-Scale-TR total**	**NSASPA total**
		**Mean ± SD**	**Mean ± SD**	**Mean ± SD**	**Mean ± SD**	**Mean ± SD**	**Mean ± SD**	**Mean ± SD**

Gender	Female	13.44 ± 3.49	13.07 ± 3.30	7.87 ± 1.86	10.48 ± 1.71	8.74 ± 1.92	53.60 ± 7.38	56.70 ± 8.46
	Male	13.15 ± 3.56	12.00 ± 3.46	7.53 ± 1.82	9.99 ± 1.86	8.48 ± 2.05	51.15 ± 7.60	53.94 ± 8.81
		*t* = 0.659 *p* = 0.511	*t* = 2.574 *p* = 0.010	*t* = 1.460 *p* = 0.145	*t* = 2.258 *p* = 0.025	*t* = 1.065 *p* = 0.287	*t* = 2.644 *p* = 0.009	*t* = 2.586 *p* = 0.010

Class level	2nd class	12.77 ± 3.13	12.16 ± 2.82	7.19 ± 1.72	9.97 ± 1.49	8.59 ± 1.73	50.69 ± 6.14	51.23 ± 8.00
	3rd class	13.69 ± 3.33	13.13 ± 3.25	7.72 ± 1.99	10.38 ± 1.81	8.71 ± 2.05	53.62 ± 7.40	56.27 ± 7.82
	4th grade	13.62 ± 4.07	13.07 ± 3.99	8.53 ± 1.54	10.76 ± 1.90	8.73 ± 2.09	54.71 ± 8.45	61.09 ± 7.26
		*F* = 2.368 *p* = 0.095	*F* = 2.901 *p* = 0.056	*F* = 14.441 *p* < 0.001	*F* = 5.222 *p* = 0.006	*F* = 0.152 *p* = 0.859	*F* = 8.450 *p* < 0.001	*F* = 41.723 *p* < 0.001

GPA	2.01–2.50	13.33 ± 4.68	14.33±,14	7.50 ± 1.97	10.50 ± 2.07	8.17 ± 2.71	53.83 ± 9.09	54.50 ± 14.60
	2.51–3.00	13.24 ± 3.28	12.24 ± 3.13	7.24 ± 2.18	10.10 ± 1.91	8.40 ± 2.02	51.21 ± 7.71	53.71 ± 6.78
	3.01–3.50	13.38±,53	12.98 ± 3.40	7.90 ± 1.81	10.40 ± 1.74	8.74 ± 1.94	53.39 ± 7.49	56.45 ± 8.64
	3.51–4.00	13.44 ± 3.55	11.44 ± 3.20	7.48 ± 1.45	10.24 ± 1.74	8.60 ± 1.87	51.20 ± 6.73	55.24 ± 9.35
		*X* ^2^ = 0.045 *p* = 0.997	*X* ^2^ = 5.809 *p* = 0.121	*X* ^2^ = 5.377 *p* = 0.146	*X* ^2^ = 1.551 *p* = 0.670	*X* ^2^ = 0.496 *p* = 0.920	*X* ^2^ = 3.293 *p* = 0.349	*X* ^2^ = 4.296 *p* = 0.231

Type of high school graduated	Anatolian High School	13.36 ± 3.56	12.96 ± 3.41	7.67 ± 1.92	10.23 ± 1.76	8.72 ± 2.01	52.93 ± 7.74	56.15 ± 8.39
	Imam Hatip High School	13.29 ± 3.54	12.14 ± 2.08	7.62 ± 1.32	10.57 ± 1.75	8.33 ± 0.86	51.95 ± 5.55	53.43 ± 6.86
	General high school	9.33 ± 4.93	13.33 ± 4.04	7.00 ± 1.73	9.67 ± 1.15	9.00 ± 1.73	48.33 ± 4.04	42.33 ± 6.35
	Technical and health education high schools	13.44 ± 3.60	11.69 ± 4.38	8.38 ± 1.67	11.06 ± 1.57	8.88 ± 1.41	53.44 ± 7.84	57.06 ± 10.82
	Other	13.67 ± 3.10	12.57 ± 3.22	8.24 ± 1.68	10.72 ± 1.81	8.50 ± 2.23	53.70 ± 7.14	56.76 ± 9.24
		*X* ^2^ = 2.590 *p* = 0.629	*X* ^2^ = 2.496 *p* = 0.645	*X* ^2^ = 6.198 *p* = 0.185	*X* ^2^ = 5.859 *p* = 0.210	*X* ^2^ = 2.124 *p* = 0.713	*X* ^2^ = 2.862 *p* = 0.581	*X* ^2^ = 8.577 *p* = 0.073

Previous training on pain management	Yes	13.56 ± 3.54	12.81 ± 3.20	7.81 ± 1.79	10.22 ± 1.82	8.55 ± 1.93	52.94 ± 7.35	55.79 ± 8.47
	No	13.22 ± 3.48	12.78 ± 3.50	7.76 ± 1.90	10.45 ± 1.71	8.76 ± 1.98	52.97 ± 7.64	56.11 ± 8.75
		*U* = 12284.50 *p* = 0.189	*U* = 13224.50 *p* = 0.826	*U* = 13398.50 *p* = 0.986	*U* = 12406.00 *p* = 0.236	*U* = 12422.50 *p* = 0.240	*U* = 13168.00 *p* = 0.776	*U* = 13225.00 *p* = 0.827

Type of pain management training	Only theoretical	13.93 ± 3.36	12.85 ± 3.31	7.69 ± 2.02	10.29 ± 1.85	8.74 ± 2.03	53.50 ± 7.62	56.13 ± 8.42
	Theoretical and practical	13.06 ± 3.40	12.58 ± 3.11	7.58 ± 1.79	10.13 ± 1.71	8.53 ± 1.89	51.88 ± 6.72	54.45 ± 8.38
		*U* = 2558.50 *p* = 0.119	*U* = 2777.50 *p* = 0.439	*U* = 2804.50 *p* = 0.495	*U* = 2837.00 *p* = 0.572	*U* = 2982.50 *p* = 0.972	*U* = 2616.50 *p* = 0.179	*U* = 2597.50 *p* = 0.158

Previous training on the management of vital signs	Yes	13.52 ± 3.55	12.67 ± 3.25	7.70 ± 1.80	10.30 ± 1.84	8.65 ± 1.85	52.84 ± 7.48	55.28 ± 8.61
	No	13.22 ± 3.48	12.91 ± 3.49	7.85 ± 1.91	10.40 ± 1.68	8.70 ± 2.06	53.08 ± 7.56	56.67 ± 8.60
		*U* = 12974.50 *p* = 0.356	*U* = 12953.50 *p* = 0.343	*U* = 12844.50 *p* = 0.275	*U* = 13157.50 *p* = 0.471	*U* = 13179.00 *p* = 0.483	*U* = 13540.50 *p* = 0.786	*t* = −1.473 *p* = 0.142

Vital signs type of training	Only theoretical	13.48 ± 4.32	12.48 ± 3.09	6.86 ± 2.17	10.10 ± 1.92	8.86 ± 2.12	51.79 ± 9.53	55.00 ± 9.22
	Theoretical and practical	13.52 ± 3.18	12.81 ± 3.20	7.71 ± 1.79	10.33 ± 1.78	8.70 ± 1.72	53.06 ± 6.66	54.92 ± 8.65
		*t* = −0.045 *p* = 0.965	*t* = −0.503 *p* = 0.615	*t* = −2.232 *p* = 0.027	*t* = −0.614 *p* = 0.540	*U* = 2058.5 *p* = 0.81	*t* = −0.684 *p* = 0.499	*t* = 0.046 *p* = 0.963

*Note: t*: independent sample *t* test, F: ANOVA test, U: Mann–Whitney *U* test, and *X* ^2^: Kruskal–Wallis test.

Abbreviations: GPA, grade point average; SD, standard deviation.

^∗^ *p* < 0.05.

The mean V‐Scale‐TR score of female nursing students (53.60 ± 7.38) was higher than that of male students (51.15 ± 7.60) (*p* < 0.05). Similarly, female students had higher mean scores in the technology (13.07 ± 3.30) and knowledge (10.48 ± 1.71) subdimensions compared to males (12.00 ± 3.46; 9.99 ± 1.86, respectively) (*p* < 0.05).

The V‐Scale‐TR total score differed significantly according to grade level (*p* < 0.05). According to Bonferroni post hoc tests, second‐year students (50.69 ± 6.14) had lower mean scores than third‐year (53.62 ± 7.40) and fourth‐year students (54.71 ± 8.45) (*p* < 0.05).

Students who received both theoretical and practical training in vital signs management had higher communication subdimension scores (7.71 ± 1.79) compared to those who received only theoretical training (6.86 ± 2.17).

No statistically significant differences were found in V‐Scale‐TR scores according to GPA, type of high school graduated, type of previous pain management training, type of pain management training, or type of vital signs training (*p* > 0.05).

The mean NSASPA score of female students (56.70 ± 8.46) was higher than that of male students (53.94 ± 8.81) (*p* < 0.05). NSASPA scores also differed significantly according to grade level (*p* < 0.05). Post hoc analysis revealed that fourth‐year students (61.09 ± 7.26) had higher scores than third‐year (56.27 ± 7.82) and second‐year students (51.23 ± 8.00), and third‐year students scored higher than second‐year students (*p* < 0.05).

No statistically significant differences were observed in NSASPA scores according to GPA, type of high school graduated, type of previous pain management training, type of pain management training, or type of vital signs training (*p* > 0.05).

### 3.4. The Relationship Between NSASPA and V‐Scale‐TR Scores Among Nursing Students

The correlation findings between NSASPA and V‐Scale‐TR and its subdimensions are presented in Table 2. The results showed a moderate positive correlation between NSASPA and V‐Scale‐TR total score (*r* = 0.464, *p* < 0.05). Regarding the subdimensions, NSASPA was moderately positively correlated with workload (*r* = 0.363, *p* < 0.05), communication (*r* = 0.421, *p* < 0.05), and knowledge (*r* = 0.253, *p* < 0.05), while a weak positive correlation was observed with technology (*r* = 0.283, *p* < 0.05). No statistically significant relationship was found between NSASPA and key indicators (*r* = 0.019, *p* = 0.731).

**TABLE 2:** Relationships between NSASPA and V‐Scale‐TR and their subdimensions in nursing students.

			V‐Scale‐TR					
			Workload	Technology	Communication	Knowledge	Key indicators	V‐Scale‐TR total
V‐Scale‐TR	Technology	*r*	0.367^∗∗^					
		*p*	*p* ≤ 0.001					
	Communication	*r*	0.025	−0.042				
		*p*	0.655	0.442				
	Knowledge	*r*	0.323^∗∗^	0.279^∗∗^	0.322^∗∗^			
		*p*	*p* ≤ 0.001	*p* ≤ 0.001	*p* ≤ 0.001			
	Key indicators	*r*	0.139^∗^	0.352^∗∗^	−0.328^∗∗^	0.042		
		*p*	0.011	*p* ≤ 0.001	*p* ≤ 0.001	0.444		
	V‐Scale‐TR total	*r*	0.750^∗∗^	0.767^∗∗^	0.229^∗∗^	0.601^∗∗^	0.413^∗∗^	
		*p*	*p* ≤ 0.001	*p* ≤ 0.001	*p* ≤ 0.001	*p* ≤ 0.001	*p* ≤ 0.001	
NSASPA total		*r*	0.363^∗∗^	0.283^∗∗^	0.421^∗∗^	0.253^∗∗^	0.019	0.464^∗∗^
		*p*	*p* ≤ 0.001	*p* ≤ 0.001	*p* ≤ 0.001	*p* ≤ 0.001	0.731	*p* ≤ 0.001

*Note: r*: Pearson correlation (*r*: 0–0.3: weak correlation; 0.3–0.7: medium correlation; 0.7–1: high correlation).

^∗^ *p* < 0.05.

^∗∗^ *p* < 0.01.

### 3.5. Multiple Linear Regression Analysis of Predictors of Nursing Students’ Attitudes Toward Pain Assessment: The Role of V‐Scale‐TR Scores

Model 1 included all demographic variables, Model 2 included the subdimensions of V‐Scale‐TR, and Model 3 included demographic variables and subdimensions of V‐Scale‐TR, as presented in Table 3. In Model 1, it was determined that gender, grade level, and the type of high school graduated from had a significant effect on nurses’ attitudes toward pain (*p* < 0.05). Demographic variables explained 24.4% of the variance in nursing students’ attitudes toward pain (*R* ^2^ = 0.244, *p* < 0.001). In Model 2, the attitude toward vital signs has a significant effect on nursing students’ attitudes toward pain, and 32.9% of the variance is explained by the subdimensions of the V‐Scale‐TR (*R* ^2^ = 0.329, *p* < 0.001). In Model 2, it was determined that workload, communication, and technology subdimensions had a significant effect on nurses’ attitudes toward pain (*p* < 0.05).

**TABLE 3:** Multiple linear regression analysis of V‐Scale‐TR and NSASPA among nursing students (*n* = 332).

Variables^a^	Model 1^b^					Model 2^c^					Model 3^d^				
	B	SE	95% CI		*p*	B	SE	95% CI		*p*	B	SE	95% CI		*p*
			Lower bound	Upper bound				Lower bound	Upper bound				Lower bound	Upper bound	
Age	0.588	0.520	−0.440	1.616	0.260						0.421	0.451	−0.472	1.314	0.353
Gender	−3.147	1.436	−5.987	−0.307	0.030						−1.731	1.263	−4.231	0.769	0.173
Class level	4.393	1.037	2.342	6.445	p ≤ 0.001						3.064	0.930	1.224	4.905	0.001
Type of high school graduated	1.078	0.402	0.282	1.873	0.008						0.623	0.363	−0.095	1.340	0.088
GPA	−0.174	1.085	−2.321	1.973	0.873						−0.035	0.947	−1.909	1.838	0.970
Previous training on the management of vital signs	−3.059	3.139	−9.268	3.150	0.332						−2.772	2.743	−8.200	2.656	0.314
Vital signs type of training	−0.485	1.974	−4.389	3.420	0.806						−2.521	1.749	−5.982	0.940	0.152
Previous training on pain management	−4.519	2.410	−9.286	0.249	0.063						−1.761	2.136	−5.988	2.466	0.411
Type of pain management training	0.113	1.514	−2.882	3.107	0.941						1.399	1.326	−1.225	4.024	0.293
Workload						0.709	0.123	0.467	0.951	p ≤ 0.001	0.635	0.178	0.284	0.987	p ≤ 0.001
Technology						0.472	0.134	0.210	0.735	p ≤ 0.001	0.542	0.198	0.150	0.934	0.007
Communication						2.124	0.238	1.656	2.592	p ≤ 0.001	1.659	0.349	0.969	2.350	p ≤ 0.001
Knowledge						−0.202	0.253	−0.700	0.296	0.426	−0.454	0.368	−1.183	0.274	0.219
Key indicators						0.287	0.225	−0.157	0.730	0.204	0.056	0.321	−0.579	0.692	0.861
*R* ^2^	0.293					0.339					0.491				
Adjusted R2	0.244					0.329					0.435				
F	6.064					33.492					8.745				
*p*	p ≤ 0.001					p ≤ 0.001					p ≤ 0.001				
VIF	1.076–1.651					1.234–1.344					1.100–1.775				
Durbin–Watson statistic	1.951					1.835					2.012				

*Note:* B, unstandardized coefficient.

Abbreviations: CI, confidence interval; SE, standard error; VIF, variance inflation factor.

^a^Missing data not included.

^b^Model 1: Sociodemographic data.

^c^Model 2: V‐Scale‐TR.

^d^Model 3: Sociodemographic data and V‐Scale‐TR.

^∗^ *p* < 0.05.

^∗∗^ *p* < 0.001.

In Model 3, 43.5% of the variance on nursing students’ attitudes toward pain is explained by demographic variables and subdimensions of the V‐Scale‐TR (*R* ^2^ = 0.435, *p* < 0.001). In this model, it was determined that class level, workload, communication, and technology subdimensions had a significant effect on nurses’ attitudes toward pain (*p* < 0.05).

The independence of error terms in regression models was examined using the Durbin–Watson statistic. The obtained values were 1.951 for Model 1, 1.835 for Model 2, and 2.012 for Model 3. The fact that these values are close to 2 indicates that there is no significant autocorrelation between the error terms in the models [23].

## 4. Discussion

The aim of this study was to examine nursing students’ attitudes toward vital signs assessment and pain according to their demographic characteristics and to identify the effect of attitudes toward vital signs on attitudes toward pain assessment and the predictors of attitudes toward pain. This study was conducted in line with this aim, and the findings are discussed accordingly. These findings may contribute to the development and implementation of educational interventions to promote positive attitudes toward vital signs and pain monitoring among future nursing students.

In this study, it was found that nursing students’ attitudes toward vital signs were at a moderate level. The results in the literature are similar to our study results. Studies conducted in Türkiye have reported that the average score on the attitude scale toward vital signs among nursing students is at a moderate level. [3, 7]. Another study conducted in Saudi Arabia reports that students’ attitudes toward life‐science monitoring are poor, which is a cause for concern [2]. In a study conducted in Serbia, it was reported that the majority of nursing students had an ambivalent attitude toward the monitoring of vital signs [24]. It is thought that the fact that nursing students’ attitudes toward vital signs are generally at a moderate level may be due to the disconnection between theoretical knowledge and clinical practice, lack of experience, and insufficient awareness of the importance of vital signs.

In this study, it was found that nursing students developed moderate attitudes toward pain assessment. In the studies conducted with nursing students in the literature, it has been observed that students have different attitudes. In the literature, studies are reporting that nursing students have incomplete attitudes toward pain [25] and low‐level attitudes [26–28], and similar to our research result, students have moderate attitudes [29, 30]. However, these two domains are both integral to comprehensive patient assessment and require similar cognitive processes such as observation, prioritization, and clinical judgment [17].

The findings of the study revealed that nursing students’ attitudes toward pain were generally at a medium or low level; this situation suggests that students have difficulty in grasping the importance of pain assessment and need more clinical guidance in this regard. It reveals the need to improve pain education programs to improve nursing students’ attitudes toward pain assessment [18].

The findings show that female nursing students have more positive attitudes toward vital signs than male students. In a study conducted in Turkey, it was reported that gender did not affect the attitude toward vital signs [7], and in a study conducted in Saudi Arabia, contrary to our research findings, male students’ attitudes toward vital signs were more positive [2]. These findings suggest that gender‐based attitudes may be affected by cultural differences and may change depending on the education system or gender roles.

In this study, it was determined that female students’ attitudes toward technology and knowledge, which are subdimensions of attitudes toward vital signs, were more positive than male students. Similar to the findings of our study, one study reported that female students scored significantly higher than male students in the knowledge subdimension [7]. In contrast to our findings, another study reported that male students had a more positive attitude toward technology and knowledge dimensions [2]. This difference may be related to the fact that social expectations regarding gender roles, access to technology, and approaches in educational processes vary from country to country; therefore, the more positive attitudes of female students in technology and knowledge dimensions can be explained by the fact that female students are encouraged more in these areas in nursing education in Turkey.

In our study, it was determined that nursing students’ attitudes toward vital signs differed according to class level, and especially the attitudes of second‐year students were lower than those of third‐ and fourth‐year students. In a study conducted in Türkiye, it was found that fourth‐year students had more positive attitudes in the communication subdimension [7]. In a study conducted in Saudi Arabia, it was found that second‐year students had weaker attitudes toward “technology” than third‐, fourth‐, and internship‐year students; second‐year students had better attitudes toward “communication” than third‐ and fourth‐year students; and their attitudes toward “workload” were better than those of fourth‐year students [2]. The lower attitudes of second‐year nursing students toward vital signs may be due to the lack of clinical experience and the intensity of the theoretical education process in this period.

In this study, it was determined that female nursing students showed more positive attitudes toward pain than male students. In a previous study conducted in Turkey, it was reported that female students had a more sensitive attitude toward pain, similar to the results of this study [31]. In another study conducted in the same country, it was reported that gender did not affect attitudes toward pain [32].

In our study, it was determined that the attitudes of nursing students toward pain differed according to the grade level, and especially the attitudes of second‐year students were lower than those of third‐year and fourth‐year students. Similar to our results, studies conducted in Turkey reported that attitudes toward pain increased with increasing grade level [30, 31]. In a study conducted in China, it was reported that different levels of education affected attitudes toward pain and that university students developed more positive attitudes [33]. In a study conducted in Canada, it was found that university‐level nursing students developed more positive attitudes toward pain [28]. In a study conducted in Hong Kong, it was reported that first‐year students had lower attitudes toward pain than third‐year students [34].

In this study, several factors were identified as important predictors of nursing students’ attitudes toward pain. Monitoring vital signs supports systematic patient observation and early recognition of changes in patient status [35]. As students develop more positive attitudes toward assessing vital signs, they may approach patient monitoring and symptom assessment with greater care [36]. Since pain is considered the fifth vital sign, this increased awareness may lead students to perceive pain assessment as a vital part of patient care rather than a subjective complaint [37, 38]. Therefore, positive attitudes toward assessing vital signs can contribute to more effective and sensitive approaches to pain assessment.

The absence of statistically significant differences in V‐Scale‐TR and NSASPA scores based on GPA, type of high school, and educational variables suggests that students’ attitudes toward vital signs and pain assessment cannot be explained solely by academic achievement or the education received. This finding indicates that factors such as clinical experience, observational learning, and frequency of practice may play a more influential role in shaping these attitudes [39]. Furthermore, the exposure of participants to similar educational content and the relatively homogeneous structure of the sample may have contributed to the absence of significant differences across these variables.

Gender, grade level, and type of high school graduated from were significant predictors of nursing students’ attitudes toward pain. These results suggest that educational experiences and clinical opportunities may affect nursing students’ attitudes toward pain assessment [18].

In this study, workload, communication, and technology subdimensions of the attitudes toward vital signs scale were significant predictors of nursing students’ attitudes toward pain. Correlation analysis also supported this finding. It is seen that nursing students’ attitudes toward pain assessment improve positively with the increase in their positive attitudes towards vital signs. Pain is considered the fifth vital sign in the literature [40]. Because this approach emphasizes that pain, like other vital signs, should be monitored regularly and treated seriously, it causes students who are sensitive to vital signs to develop more conscious and responsible attitudes in pain assessment. The findings of this research make it significant that nursing students’ attitudes toward vital signs directly affect their attitudes toward pain.

## 5. Limitations of the Study

This study is important in terms of contributing to the limited literature aiming to compare nursing students’ attitudes toward vital signs and pain. The study was conducted in a public university in Turkey, and the generalizability of our study in provinces and countries with different nursing education approaches is limited. Therefore, it is recommended that the study be repeated in different sample groups and in different cultures. Data were collected through a self‐report questionnaire method. This may have caused students to respond in line with social bias and hide their true attitudes.

## 6. Conclusion

This study shows a significant and reciprocal relationship between nursing students’ attitudes toward vital signs and pain assessment, suggesting that these competencies develop together throughout their education. The findings also show that factors such as gender, academic level, workload, communication, and technology influence these attitudes. Based on these results, integrating vital sign and pain assessment within the same educational activities (such as case‐based learning or simulation) and including combined elements in OSCEs may better reflect clinical practice and support skill development. Considering the impact of communication and workload, adding structured communication training and workload management strategies into courses may further improve students’ clinical decision‐making. Overall, the findings emphasize the importance of connecting theoretical education with clinical practice through integrated, practice‐based approaches. Future research may focus on longitudinal and intervention studies using objective measures and technology‐supported learning to further examine these competencies.

## Author Contributions

Burcu Nal: conceptualization, methodology, software, formal analysis, writing–original draft, writing–review and editing, and supervision.

Necibe Dağcan Şahin: conceptualization, methodology, software, formal analysis, writing–original draft, and writing–review and editing.

Gamze Ünver: conceptualization and writing–review and editing.

## Funding

No funding was received for this manuscript.

## Ethics Statement

Permission was obtained from the Kütahya Health Science University’s nursing department (decision letter dated 12.03.2025 and numbered 177515) and the university’s non‐interventional clinical research ethics committee (decision dated 27.02.2025 and numbered 2025/03–02). Permission was obtained from the authors for the use of the V‐Scale‐TR and Nursing Students’ Attitudes Towards Pain Scale.

## Consent

Written informed consent was obtained from all student participants before participation in the study.

## Conflicts of Interest

The authors declare no conflicts of interest.

## Data Availability

The data that support the findings of this study are available from the corresponding author upon reasonable request.
